# Uric Acid and Impulse Control Disorders in Parkinson’s Disease: A Cross-Sectional Analysis

**DOI:** 10.3390/medicina61101789

**Published:** 2025-10-03

**Authors:** Mateusz Toś, Agata Dymek, Agata Morka, Paulina Włodarczyk, Joanna Siuda

**Affiliations:** 1 Department of Neurology, Faculty of Medical Sciences in Katowice, Medical University of Silesia, 40-055 Katowice, Poland; mateusz.tos@sum.edu.pl (M.T.);; 2 Regional Specialist Hospital No. 4 in Bytom, 41-902 Bytom, Poland; 3 Students’ Scientific Association, Department of Neurology, Faculty of Medical Sciences in Katowice, Medical University of Silesia, 40-055 Katowice, Poland

**Keywords:** impulse control disorder, Parkinson’s disease, uric acid, uric acid/creatine ratio, hypersexuality, pathological gambling, impulsivity

## Abstract

*Background and Objectives:* Impulse control disorders (ICDs) are frequent non-motor complications of Parkinson’s disease (PD), usually related to dopaminergic therapy. Uric acid (UA) has been studied as a biomarker of PD severity and has been linked to impulsivity in non-PD populations. However, its association with ICDs in patients with PD (PwPs) has not been investigated. This study aimed to assess the relationship between serum UA levels, the uric acid to creatinine ratio (UA/Cr), and ICD prevalence in PwPs. *Materials and Methods:* We enrolled 172 PwPs hospitalized for follow-up or treatment modification. ICDs were screened with the Questionnaire for Impulsive-Compulsive Disorders in Parkinson’s Disease (QUIP). Clinical data included demographics, disease severity, motor and non-motor symptoms, and dopaminergic treatment. Fasting serum UA and UA/Cr were determined. *Results:* ICDs were present in 24.42% of patients, most commonly binge eating and compulsive buying. PwPs with ICDs had longer disease duration, more motor complications, higher dopaminergic doses, and more frequent dopamine agonist use. No relationship was found between absolute UA and overall ICD occurrence. However, lower UA/Cr was observed in patients with hypersexuality and pathological gambling, as well as in those with multiple ICD subtypes. Logistic regression confirmed that higher UA/Cr reduced the odds of hypersexuality (OR = 0.55; 95% CI 0.31–0.98) and multiple ICDs (OR = 0.33; 95% CI 0.13–0.84). As a secondary finding, lower absolute UA was observed in PwPs with more advanced motor symptoms, motor complications, depressive symptoms, and cognitive impairment. *Conclusions:* Lower UA/Cr was selectively associated with specific ICD subtypes and with the coexistence of multiple ICDs in patients with PD. UA/Cr may serve as a marker of ICD heterogeneity. Confirmation in larger, prospective cohorts is needed to establish clinical relevance.

## 1. Introduction

Parkinson’s disease (PD) is the second most common neurodegenerative disorder worldwide, following Alzheimer’s disease (AD) [1]. Its pathogenesis is primarily attributed to the progressive degeneration of dopaminergic neurons in the substantia nigra pars compacta of the midbrain. The core clinical features, which form the basis of the diagnostic criteria for PD, include bradykinesia, muscular rigidity, and resting tremor [2]. In addition to these motor symptoms, PD is characterized by a wide range of non-motor symptoms (NMS) that significantly impair the quality of life of both patients and their caregivers [3]. Non-motor symptoms encompass cognitive, cardiovascular, autonomic, and sleep disturbances, as well as impulse control disorders (ICDs) [4,5,6]. These psychiatric conditions are often underrecognized, yet they exert a substantial impact on social functioning. ICDs are defined by impaired behavioral control and an inability to resist potentially harmful urges [7]. Such behaviors are typically preceded by a sense of mounting tension and followed by feelings of relief or gratification. The most commonly reported ICDs include pathological gambling, hypersexuality, binge eating, and compulsive shopping [8,9]. Less frequent manifestations described in the literature comprise reckless driving, trichotillomania, compulsive smoking, and excessive charity [10]. ICD-related behaviors (ICD-RB) may also occur, including punding (the compulsive repetition of purposeless activities), hobbyism, hoarding, and excessive aimless wandering (walkabout). Patients with ICDs frequently lack full awareness of the consequences of their behaviors or experience feelings of shame, leading them to conceal these behaviors [11]. The development of ICDs in PD is primarily linked to dopaminergic replacement therapy (DRT), particularly the use of dopamine agonists (DA) [12,13].

### Uric Acid and Its Role in PD Pathophysiology

As research efforts intensify toward identifying new therapeutic strategies and early diagnostic biomarkers for PD, numerous studies have explored the relationship between disease progression and the concentration of various biomarkers in serum and other biological materials [14,15]. One of the most extensively investigated biomarkers is serum uric acid (UA), a naturally occurring antioxidant. Multiple studies have demonstrated an association between low UA levels and an increased risk of developing PD [16,17,18,19]. Furthermore, some researchers have proposed that UA may serve as a predictive biomarker for the clinical course of the disease, given the documented correlation between lower UA concentrations and more severe disease manifestations [20]. It has also been reported that serum UA levels gradually decline over the course of PD. Additionally, based on available clinical data, despite certain inconsistencies, many authors suggest that reduced UA levels may be significantly associated with the severity of NMS in PD [21,22]. From a pathophysiological perspective, oxidative stress and neuroinflammation are considered central mechanisms driving dopaminergic neurodegeneration in PD [23,24]. Excessive mitochondrial production of reactive oxygen species (ROS) leads to lipid peroxidation, protein and DNA damage, and ultimately neuronal death, while microglial activation maintains a chronic pro-inflammatory environment that accelerates these processes [23]. UA, as one of the most potent endogenous antioxidants in plasma and cerebrospinal fluid, can scavenge peroxynitrite and other ROS, thereby stabilizing redox homeostasis and modulating immune responses [25]. Consequently, reduced UA may impair antioxidant defenses, promote oxidative and nitrosative stress, and increase neuronal vulnerability, providing a biological rationale for its association with both PD risk and severity [25,26]. Independent of its role in PD pathophysiology, UA has also been examined in relation to impulsivity across different populations. Available evidence indicates a significant association between UA levels and impulsivity, both in the general population and among individuals with psychiatric disorders [27,28].

To date, no studies have directly evaluated the relationship between serum UA levels and the occurrence of ICD in PwPs. Considering, on one hand, the proposed neuroprotective role of UA in the course and severity of PD, and on the other hand, reports suggesting a link between higher UA levels and increased impulsivity, the aim of the present study was to investigate the potential association between UA levels and ICD prevalence in PwPs.

## 2. Materials and Methods

### 2.1. Participant Characteristics and Data Collection Procedure

The study included 172 PwPs hospitalized at the Department of Neurology, University Clinical Center in Katowice, Poland, for planned follow-up or treatment modification. The study group was selected from the cohort described by Toś et al., based on predefined inclusion and exclusion criteria [29]. The inclusion criteria were: (a) clinically confirmed PD according to the Movement Disorder Society Clinical Diagnostic Criteria for Parkinson’s Disease [30], and (b) a clinical condition allowing the patient to complete questionnaires. The exclusion criteria were: (a) diagnosis of a parkinsonian syndrome other than PD, (b) advanced dementia syndrome, and (c) use of medications affecting UA levels (including allopurinol, febuxostat, thiazide and loop diuretics, tacrolimus, cyclosporine, and cytotoxic drugs). Given the prospective design, the study protocol was approved by the Bioethics Committee of the Medical University of Silesia (decision number PCN/0022/KB1/99/I/19/21). The study was conducted in accordance with the ethical principles outlined in the Declaration of Helsinki (1F4) and its subsequent amendments. Demographic and clinical information (age, sex, symptom onset, PD duration, and treatment history) was obtained from a review of medical records and structured patient interviews.

### 2.2. Study Procedures and Assessment Tools

The prevalence of ICD among PwPs was assessed using the Polish translation of the Questionnaire for Impulsive-Compulsive Disorders in Parkinson’s Disease (QUIP), obtained from the University of Pennsylvania. QUIP is a self-administered screening instrument completed by both the patient and caregiver to assess the presence of ICD [31]. It consists of three sections, each beginning with a brief description of the respective disorder, followed by specific items addressing individual ICDs and ICD-RBs. Cut-off scores were applied in accordance with the original validation study: (a) pathological gambling ≥ 2 affirmative responses; (b) hypersexuality ≥ 1; (c) compulsive buying ≥ 1; (d) binge eating ≥ 2 affirmative responses. It should be noted that QUIP is a screening tool with high sensitivity but limited specificity. Positive results were not verified with structured psychiatric interviews or additional clinical confirmation; therefore, findings reflect screening-positive cases rather than clinically confirmed diagnoses

In each participant, fasting blood samples were obtained to determine UA concentration and the uric acid to creatinine ratio (UA/Cr). Reference ranges for UA concentration were adopted from the local laboratory standards (2.4–5.70 mg/dL). The UA/Cr ratio is considered by many researchers to better reflect the biological availability of UA in the body than absolute serum UA levels, as it accounts for renal function as a modulating factor [32,33].

Disease severity was assessed by neurologists experienced in neurodegenerative disorders using the modified Hoehn and Yahr (H&Y) scale and the Polish version of the Movement Disorder Society–Unified Parkinson’s Disease Rating Scale Part III (MDS-UPDRS Part III), evaluated in both the “OFF” and “ON” states [34,35]. Mood disturbances were assessed by a trained psychologist using the Polish adaptation of the Beck Depression Inventory-II (BDI-II), whereas cognitive performance was evaluated with the Mini-Mental State Examination (MMSE) and the Addenbrooke’s Cognitive Examination III (ACE-III) [36,37,38]. The levodopa equivalent daily dose (LEDD) was calculated for the total DRT, as well as separately for levodopa and DAs [39].

### 2.3. Statistical Analysis

All analyses were performed using Statistica 13.0 software (TIBCO Software Inc., Palo Alto, CA, USA). The distribution of quantitative variables was assessed with the Shapiro–Wilk test. For normally distributed data, descriptive statistics were presented as mean and standard deviation (SD), and group comparisons were performed with the independent samples Student’s *t*-test. For non-normally distributed variables, the median and interquartile range (IQR) were reported, and comparisons between two groups were conducted using the Mann–Whitney U test. For comparisons involving more than two groups with non-normally distributed variables, the Kruskal–Wallis test (non-parametric ANOVA) was applied. Categorical variables were compared using the chi-square test or Fisher’s exact test, depending on sample size. Associations between numerical variables were evaluated with Spearman’s rank correlation coefficient (R). To further explore the relationship between UA/Cr and ICDs, logistic regression analyses were conducted. Three models were fitted with the following outcomes: (1) presence of more than one ICD subtype, (2) hypersexuality, and (3) pathological gambling. All models were adjusted for age, disease duration, and dopaminergic medication dose (LEDD). Regression results were reported as odds ratios (ORs) with 95% confidence intervals (CIs). To account for multiple testing across the three models, false discovery rate (FDR) correction using the Benjamini–Hochberg method was applied. The level of statistical significance was defined as *p* < 0.05.

## 3. Results

The analysis included 172 PwPs derived from the study by Toś et al., after excluding those receiving medications affecting UA levels. Males accounted for 62.21% of the cohort. The mean age was 63.72 years (SD = 9.72), and the mean age at diagnosis was 54.07 years (SD = 10.82). Detailed clinical characteristics are presented in Table 1. Most patients were treated with a combination of levodopa and DA (56.73%), followed by levodopa monotherapy (31.58%) and DA monotherapy (5.85%). At the time of assessment, nine patients (5.26%) were not receiving DRT.

### 3.1. Prevalence of Impulse Control Disorders and Their Clinical Correlates

ICD were identified in 24.42% of patients, with 7.56% meeting criteria for at least two ICD subtypes. The most frequent ICD was binge eating (11.05%), followed by compulsive buying (10.47%) and hypersexuality (8.72%), while pathological gambling was the least common (4.65%). In addition, across the entire cohort, 20.93% of participants exhibited punding, 18.02% hobbyism, and 5.23% walkabout.

There was no significant association between ICD prevalence and sex, age, or age at diagnosis. However, PwPs with ICD had more advanced disease severity according to the H&Y scale (median 3 in both groups; *p* = 0.029), with statistical significance attributable to differences in the distribution of ranks rather than the median values themselves. No significant differences were observed in disease severity assessed with the MDS-UPDRS Part III, either in the OFF or ON medication states. Compared to non-ICD patients, those with ICD had a longer disease duration (median 11 years, IQR 8 vs. median 8 years, IQR 9; *p* = 0.004) and were more likely to experience motor complications such as levodopa-induced dyskinesia and the wearing-off phenomenon (83.33% vs. 52.31%; *p* < 0.001).

In terms of treatment, PwPs with ICD received higher doses of DRT expressed as LEDD (median 1180 mg, IQR 956 vs. median 980 mg, IQR 985; *p* = 0.038) and were more likely to use DA (83.88% vs. 55.38%; *p* = 0.001). They were also more frequently treated with a combination of levodopa and DA (76% vs. 49%; *p* = 0.031). A comparison between PwPs with and without ICD is presented in Table 2.

### 3.2. Uric Acid and Its Clinical Correlates

In the study group, the mean UA level was 5.09 mg/dL (SD = 1.49) and the mean UA/Cr ratio was 5.72 (SD = 1.36). UA levels were negatively correlated with disease severity as assessed by the H&Y scale (R = –0.28; *p* < 0.001) and by the MDS-UPDRS Part III in the OFF state (R = –0.27; *p* = 0.004). UA levels were significantly higher in men than in women (median 5.37, IQR 1.54 vs. median 4.56, IQR 1.34; *p* = 0.001). PwPs with motor complications had lower UA levels compared with those without motor complications (median 4.61, IQR 1.57 vs. median 5.38, IQR 1.39; *p* = 0.002). Similarly, lower UA levels were observed in patients with more severe depressive symptoms (*p* = 0.029) and in those with cognitive impairment (*p* = 0.035). PwPs with motor complications had a lower UA/Cr ratio than those without (median 5.23, IQR 1.82 vs. median 6.28, IQR 1.38; *p* = 0.013) and this ratio was also lower in patients with cognitive impairment (*p* = 0.034). No other significant associations were observed between UA/Cr and sex or disease severity.

### 3.3. Uric Acid and the Occurrence of Impulse Control Disorders

**Figure 1:**
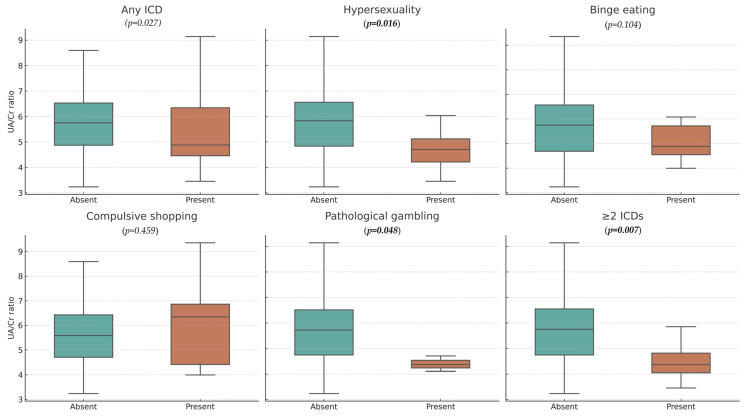
UA/Cr ratio in PwPs with and without ICD and their subtypes. Bolded *p*-values indicate statistically significant differences. UA/Cr—Uric acid/Creatine; PwPs—Patients with Parkinson’s disease; ICD—Impulse control disorder.

No association was found between serum UA levels and the overall prevalence of ICDs or its specific subtypes: pathological gambling, hypersexuality, compulsive buying, and binge eating (all *p* > 0.05). Similarly, no significant relationship was observed between the UA/Cr ratio and overall ICD occurrence (*p* = 0.278). When analyzing individual ICD subtypes, PwPs with pathological gambling had significantly lower UA/Cr ratios compared with those without this behavior (median 4.36, IQR 0.60 vs. median 5.72, IQR 1.77; *p* = 0.048). A similar association was observed in patients with hypersexuality (median 4.71, IQR 1.15 vs. median 5.83, IQR 1.77; *p* = 0.016) and in those meeting criteria for more than one ICD subtype (median 4.36, IQR 0.60 vs. median 5.72, IQR 1.77; *p* = 0.048). No significant associations between the UA/Cr ratio and binge eating or compulsive buying were found. These relationships are illustrated in Figure 1.

### 3.4. Logistic Regression Analysis

To further investigate the association between the UA/Cr ratio and ICDs occurrence, three separate logistic regression models were performed. In unadjusted analyses, higher UA/Cr ratio was associated with a 67% reduction in the odds of having multiple ICD subtypes (OR = 0.33, 95% CI 0.13–0.84, *p* = 0.020) and a 45% reduction in the odds of hypersexuality (OR = 0.55, 95% CI 0.31–0.98, *p* = 0.043). For pathological gambling, a similar trend was observed but without statistical significance (OR = 0.57, 95% CI 0.27–1.21, *p* = 0.146). In multivariable models adjusted for age, disease duration, and dopaminergic medication dose (LEDD), the protective associations remained consistent and even stronger, corresponding to an 85% reduction in the odds of multiple ICDs (OR = 0.15, 95% CI 0.03–0.75, *p* = 0.021) and a 63% reduction in the odds of hypersexuality (OR = 0.37, 95% CI 0.14–0.96, *p* = 0.040). A similar but non-significant trend was again found for pathological gambling (OR = 0.05, 95% CI 0.00–3.73, *p* = 0.176). After correction for multiple testing using the false discovery rate (FDR), the associations with multiple ICDs and hypersexuality remained at the threshold of significance (q = 0.061). The wide confidence intervals, especially for pathological gambling, underscore the limited statistical power of subgroup analyses.

## 4. Discussion

The aim of this study was to assess the association between serum UA levels and the UA/Cr ratio with the prevalence of ICD and their subtypes in PwPs. To the best of our knowledge, this is the first study to address this relationship.

ICDs were identified in about one-quarter of PwPs, a prevalence similar to that reported in our earlier studies (Toś et al., 2023; Toś et al., 2024) and consistent with data from other European and international cohorts [29,40,41,42,43]. The most frequent subtypes were binge eating and compulsive buying, whereas pathological gambling was uncommon. As previously described, ICD occurrence was associated with longer disease duration, greater H&Y severity, motor complications, higher LEDD, and particularly DA use, especially in combination therapy [44,45]. These observations are in line with both our prior findings and global literature, and are only briefly summarized here, as their detailed discussion has been provided elsewhere.

### 4.1. Uric Acid and Its Association with Parkinson’s Disease Symptoms

UA is the end product of purine catabolism and an informative biomarker of metabolic processes. Its serum concentration is influenced by genetic factors, renal function, hydration, diet, and comorbidities [46]. Accordingly, we examined both absolute UA and the UA/Cr ratio, which partially adjusts for renal function.

Oxidative stress and neuroinflammation are fundamental, mutually reinforcing mechanisms in the development of neurodegenerative diseases [47]. An imbalance in redox homeostasis leads to excessive production of reactive oxygen species (ROS), primarily in the mitochondria, resulting in lipid peroxidation, DNA and protein damage, and neuronal degeneration [48,49,50]. Neuropathological studies have demonstrated pronounced oxidative damage in the brains of patients with PD, as well as AD, multiple sclerosis (MS), and mild cognitive impairment (MCI), including mitochondrial dysfunction and myelin injury [51]. At the same time, neurodegeneration is accompanied by a significant decline in blood levels of non-enzymatic antioxidants, including UA [52]. Neuroinflammation further amplifies these processes: oxidative stress activates signaling pathways that increase the expression of pro-inflammatory genes, while immune cells release additional reactive oxygen species, aggravating the damage. This bidirectional interaction between oxidative stress and inflammation accelerates neuronal degeneration and the progression of neurodegenerative diseases [53].

In the context of PD pathogenesis, there is growing interest in the role of UA as a potential neuroprotective factor. Numerous studies over the past years have suggested that higher UA levels may be associated with slower progression of PD, a milder clinical phenotype, and even a reduced risk of developing the disease [54,55,56]. While our findings are in partial agreement with previous evidence, they should be interpreted with caution, as the cross-sectional design of the study precludes any causal inference. The associations between lower UA levels and greater disease severity observed in our cohort do not establish a protective role of UA but rather indicate correlations that require validation in prospective investigations. Specifically, in PwPs with greater disease severity as measured by the H&Y scale, and in those with more pronounced motor symptoms as assessed by the MDS-UPDRS Part III in the “OFF” state, we observed lower absolute UA levels, which supports the hypothesis that UA exerts a protective effect. Moreover, lower UA and UA/Cr values were also identified in patients experiencing motor complications of PD, such as wearing-off phenomena and levodopa-induced dyskinesias, conditions typically associated with more advanced stages of the disease. The mechanisms underlying the putative neuroprotective effect of UA in PwPs are not fully elucidated. The most widely accepted explanation highlights the antioxidant capacity of UA, which enables it to neutralize reactive oxygen species and thereby limit oxidative stress [57,58,59]. This aspect is of particular importance in PD, where oxidative stress is considered a central contributor to dopaminergic neurodegeneration [59,60,61]. A reduction in UA levels may thus impair antioxidant defense capacity, creating a pro-oxidant environment that increases neuronal vulnerability. In this way, insufficient UA could facilitate the degeneration of dopaminergic neurons and contribute to the progression of both motor and non-motor symptoms observed in PD. In drug-naive PwPs, UA levels correlated with the severity of dopaminergic impairment in the caudate, putamen, and striatum, as assessed by dopamine transporter imaging [62].

NMS, alongside motor symptoms, have a substantial impact on the daily functioning of PwPs [63,64]. The strongest effects on quality of life have been described for constipation, urinary dysfunction, olfactory loss, and mood disturbances such as depression and apathy. In most available studies, NMS, which may emerge already in the prodromal phase, occurred more frequently and were more severe in patients with lower UA [5,65,66]. Studies using standardized assessment tools such as the Non-Motor Symptoms Scale (NMSS) and the Non-Motor Symptoms Questionnaire (NMS-Quest) have confirmed a significant correlation between reduced UA and the presence and severity of non-motor symptoms [67,68,69]. In our cohort, lower UA was observed in PwPs with more pronounced depressive symptoms, which supports the hypothesis that UA may also contribute to this NMS. Additional support comes from reports showing lower UA in disorders such as depression, bipolar disorder, and social anxiety [70,71,72].

A large proportion of NMS in PwPs comprises cognitive dysfunction, including memory impairment. According to available analyses, dementia may develop in up to 80% of PwPs over the course of the disease [73,74,75,76]. In our study, lower UA and UA/Cr were found in PwPs with more severe cognitive impairment, which is partially consistent with prior reports. However, existing studies remain inconclusive and do not establish a direct relationship between UA and cognitive impairment. Moccia et al. reported an association with the presence and progression of cognitive deficits in PwPs; however, this was not captured by MMSE, likely due to the test’s limited sensitivity to early disturbances in domains such as attention and memory [22,77]. Pan et al. reported an association only in men, whereas Huang et al. indicated a possible correlation between UA and Montreal Cognitive Assessment (MoCA) scores, without consistent confirmation across domain-specific tests [78,79].

Another dimension of the multifactorial role of UA concentration in the pathogenesis of various disorders, observed in populations without Parkinson’s disease, is its potential impact on impulsivity. Impulsivity is defined as a predisposition to rapid, unplanned reactions to internal or external stimuli without consideration of potential negative consequences [80]. According to Sutin et al., in the general population higher UA levels are positively correlated with a range of impulsivity-related traits, including a greater tendency to act without reflection, heightened sensation seeking, and reduced self-control [27]. Similar associations have been observed in clinical populations: individuals with antisocial personality features and those with substance use disorders demonstrated higher UA concentrations compared to control groups, with UA levels showing significant correlations with the severity of impulsivity as measured by standardized diagnostic instruments [81,82]. Supporting evidence also comes from experimental animal studies, in which artificially elevated UA levels were found to intensify exploratory activity and impulsive responses. These findings indicate that UA may influence behavioral regulation and suggest the presence of biological mechanisms underlying this relationship [27]. From an evolutionary perspective, UA has been hypothesized to serve as an activator of adaptive behaviors related to resource seeking and risk-taking, which in turn could account for its association with impulsivity traits [27,83].

Impulse control is most commonly assessed through experimental tasks targeting two domains: impulsive action (difficulty inhibiting a prepotent response) and impulsive choice (difficulty making balanced decisions that consider costs and benefits) [84]. Although research on impulsivity in PD is growing, integrative analyses across these domains remain scarce. The observed differences may reflect distinct neurodegenerative mechanisms: impulsive action primarily depends on the dorsal striatum and motor-related regions, which undergo early degeneration, whereas impulsive choice relies on the ventral striatum and prefrontal cortex, which are relatively preserved in the early stages of PD [85,86,87].

Considering that impulsivity and ICDs, although related, represent distinct entities, it seems important to investigate factors that may influence both domains simultaneously [88]. Uric acid may serve as such a candidate, given its suggested neuroprotective role in PD and its previously reported associations with impulsivity in non-PD populations. Identifying biological modulators that bridge these two phenomena could help explain the heterogeneity of previous findings and support a more integrated understanding of how trait impulsivity and clinically manifest ICDs emerge in PD.

In our cohort, we found no overall association between UA or the UA/Cr ratio and the prevalence of ICD. However, when specific ICD phenotypes were considered, PwPs with pathological gambling or hypersexuality showed lower UA/Cr values than patients without these behaviors, and a similar pattern was observed in individuals meeting criteria for two or more distinct ICDs. These differences were further supported by multivariable logistic regression models adjusted for age, disease duration, and dopaminergic medication dose, which indicated a protective association of higher UA/Cr ratio with hypersexuality and multiple ICDs. For pathological gambling, a similar direction was observed but without statistical significance. After applying false discovery rate correction for multiple testing, the associations with hypersexuality and multiple ICDs were attenuated to a trend level. Taken together, these results suggest that UA/Cr may play a protective role in specific ICD phenotypes, although the wide confidence intervals, particularly for pathological gambling, reflect the limited statistical power and warrant cautious interpretation. Rather than providing evidence that UA/Cr truly protects against ICDs, these observations point to possible associations that may differ across ICD subtypes.

To our knowledge, no previous studies have directly examined UA or UA/Cr in relation to ICD in PwPs, which limits direct comparisons with existing literature. In the broader context of sexual dysfunction, Barassi et al. linked UA to erectile dysfunction and proposed endothelial dysfunction as a possible mechanism, indirectly connecting metabolic status to sexual behaviors [89]. Extending this perspective, UA has also been associated with hormonal and neurochemical pathways relevant to impulse control, including correlations with testosterone, cortisol, norepinephrine, and serotonergic signaling [80,90,91,92,93] systems are central to arousal, reward processing, and inhibition, and their modulation may help explain why hypersexuality and gambling, rather than binge eating or compulsive buying, showed the strongest associations in our cohort. Endothelial dysfunction and reduced nitric oxide bioavailability provide an additional vascular pathway through which UA could interact with frontostriatal networks [94,95,96]. These hypotheses remain speculative and require empirical verification, particularly as the cross-sectional design of our study does not allow conclusions about causality [90].

Our results should be viewed as exploratory. Larger, multicenter studies with repeated UA and UA/Cr measurements and multivariable models are needed to assess the independence of the observed associations and differences across ICD subtypes. Such verification may improve understanding of the pathogenesis of specific ICDs in PwPs and, in the longer term, support more precise clinical management. Although speculative, these observations raise the possibility that, if confirmed in prospective studies, the UA/Cr ratio might have potential as a simple biomarker to help identify patients at increased risk of specific ICD phenotypes during dopaminergic therapy, thereby supporting more individualized monitoring and early intervention. Given the cross-sectional design and other methodological constraints, however, these findings should be interpreted with caution, as discussed in the Strengths and Limitations Section.

### 4.2. Strengths and Limitations

To the best of our knowledge, this is the first study to assess the potential impact of serum UA concentration on the prevalence of ICD and their subtypes in PwPs. The analysis incorporated both absolute serum UA concentration and the UA/Cr ratio, which allowed for partial adjustment for the influence of renal function and increased the reliability of the conclusions. Importantly, patients receiving medications known to affect UA levels were excluded, further reducing potential bias. Another strength is the comprehensive clinical characterization of the study group, including a broad range of demographic data, detailed clinical parameters, motor complications, depressive symptoms, and precise treatment information, including LEDD for both total DRT and its individual components. Validated and widely applied assessment tools were used, including QUIP, H&Y, MDS-UPDRS, and BDI-II, which enhances comparability with other studies.

Several limitations should also be acknowledged. Two constraints merit emphasis: the single-center design, which may restrict generalizability beyond our referral population, and the absence of a non-PD control group. Conducting the study at a single tertiary referral center may have resulted in population enriched with more advanced or complex cases, thus limiting generalizability. The relatively small sample size may have reduced the statistical power, particularly for less common ICD subtypes. ICD prevalence was assessed using a single administration of the QUIP, a highly sensitive but not diagnostic instrument. The lack of psychiatric or structured clinical confirmation entails a risk of false-positive results and may have led to an overestimation of prevalence. Our findings should be interpreted as reflecting the proportion of patients at risk for ICDs rather than the true prevalence of clinically diagnosed disorders. Another limitation is that serum UA was measured only once, although UA/Cr partially adjusted for renal function. Single measurement may be influenced by diet, alcohol consumption, and lifestyle, and the absence of repeated assessments limits evaluation of parameter stability. This is particularly important, as repeated measurements would have strengthened the robustness of our findings. The absence of a non-PD control group is another limitation, as it makes it difficult to determine whether the observed UA/Cr differences are specific to Parkinson’s disease or reflect general variability related to age, sex, renal function, diet, or comorbidities. Moreover, while we discussed the potential antioxidant properties of UA, no direct markers of oxidative stress were assessed, which restricts interpretation of this proposed mechanism. Finally, as this was a cross-sectional study, causality cannot be inferred. The observed associations should not be interpreted as evidence of a protective effect of UA/Cr but rather as hypothesis-generating findings that require prospective confirmation.

## 5. Conclusions

This cross-sectional analysis of 172 PwPs identified a subtype-specific association between lower UA/Cr and hypersexuality, pathological gambling, and the coexistence of multiple ICDs. UA/Cr was more informative than absolute UA for these behavioral associations and also clustered with greater motor severity, motor complications, depressive symptoms, and cognitive impairment, lending biological plausibility to the findings. Together, these results refine the characterization of ICD heterogeneity in PD and should be regarded as exploratory, hypothesis-generating findings rather than evidence of causality. The absence of a non-PD control group and the cross-sectional design further limit causal inference. Based on our findings, we propose the following testable hypotheses for future research: (1) higher UA/Cr ratio predicts a lower risk of developing hypersexuality or multiple ICD subtypes under dopaminergic therapy; (2) repeated longitudinal measurements of UA/Cr can identify patients at increased risk of specific ICD phenotypes before their clinical onset; and (3) interventions that modify UA/Cr levels may influence the trajectory or severity of certain ICDs in patients with PD. Prospective, adequately powered, multi-center studies with repeated UA and UA/Cr measurements and detailed treatment profiling are warranted to determine whether these associations are disease-specific and to clarify their potential clinical relevance.

## Figures and Tables

**Table 1:** Demographic and clinical characteristics of the cohort.

Clinical Features (N = 172)	Treatment and LEDD
Sex: male 107 (62.21%); female 65 (37.79%)	Levodopa: 148 (86.05%)
Age (years): 63.72 ± 9.72	DA (any): 107 (62.21%)
Age at diagnosis, (years): 54.07 ± 10.82	Pramipexole: 16 (9.30%)
Disease duration, (years): 9.74 ± 6.02	Ropinirole: 85 (49.42%)
H&Y: median 3.00 (IQR 2–3)	Piribedil: 6 (3.49%)
MDS-UPDRS part III OFF: 42.06 ± 18.88	Amantadine: 47 (27.33%)
MDS-UPDRS part III ON: 20.73 ± 12.84	MAO-B inhibitors: 32 (18.60%)
Motor complications: 103 (59.88%)	COMT inhibitors: 8 (4.66%)
Levodopa–induced dyskinesia: 81 (47.09%)	Total LEDD (mg): 1104.85 ± 678.69
Wearing-off phenomenon: 86 (50.00%)	LD LEDD (mg): 911.39 ± 540.76
Cognitive status: lack of dementia 96 (55.81%), MCI 58 (33.72%), mild 16 (9.30%), moderate 2 (1.16%)	DA LEDD (mg): 212.59 ± 209.29
Depression: minimal 145 (84.30%), mild 20 (11.63%), moderate 7 (4.07%)	Therapy type: levodopa + DA 95 (56.73%); levodopa mono 56 (31.58%); DA mono 10 (5.85%); no DRT 9 (5.26%); other 1 (0.58%)

Values are presented as n (%) for categorical variables and as mean ± SD or median (IQR) for continuous variables. Abbreviations: DA—dopamine agonist; LEDD—levodopa equivalent daily dose; LD—levodopa; DRT—dopaminergic replacement therapy; MDS-UPDRS—MDS Unified Parkinson’s Disease Rating Scale; H&Y—Hoehn and Yahr; MCI—mild cognitive impairment.

**Table 2:** Clinical features associated with ICDs in patients with Parkinson’s disease.

	ICD Patients	Non-ICD Patients	*p*
Male	29 (69.05%)	78 (60%)	0.293
Age at examination (years)	67.5 (IQR 13)	65 (IQR 13)	0.136
Age of onset (years)	53.98 ± 10.16	54.11 ± 11.19	0.946
Disease duration (years)	11 (IQR 8)	8 (IQR 9)	0.004 *
MDS-UPDRS part III in OFF state	40 (IQR 22)	40 (IQR 24)	0.992
MDS-UPDRS part III in ON state	19.5 (IQR 19.5)	20 (IQR 17)	0.945
H&Y	3 (IQR 1)	3 (IQR 1)	0.029 *
Motor fluctuations	35 (83.33%)	68 (52.31%)	<0.001 *
DA use	35 (83.33%)	72 (55.38%)	<0.001 *
Levodopa	39 (93.33%)	109 (84.67%)	0.168
MAO-B inhibitors	11 (26.19%)	21 (16.15%)	0.146
Amantadine use	13 (30.95%)	34 (26.15%)	0.544
DA-LEDD	160 (IQR 83)	160 (IQR 183)	0.433
LD-LEDD	950 (IQR 825)	800 (IQR 700)	0.119
Total LEDD	1180 (IQR 956)	980 (IQR 985)	0.038 *

Values are presented as absolute numbers (n) with percentages (%) for categorical variables and as mean ± SD or median (IQR) for continuous variables. Abbreviations: *—statistically significant differences; ICD—impulse control disorder; DA—dopamine agonist; LEDD—levodopa equivalent daily dose; LD—levodopa; MDS-UPDRS—MDS Unified Parkinson’s Disease Rating Scale; H&Y—Hoehn and Yahr.

## Data Availability

The datasets generated and analyzed during the current study are available on request from the corresponding author. The data are not publicly available due to privacy reasons.

## Abbreviations

The following abbreviations are used in this manuscript:

ACE-III	Addenbrooke’s Cognitive Examination III
AD	Alzheimer’s disease
BDI-II	Beck Depression Inventory-II
CI	Confidence interval
DA	Dopamine agonist
DRT	Dopaminergic replacement therapy
H&Y	Hoehn and Yahr scale
ICD	Impulse control disorder
ICD-RB	Impulse control disorder-related behavior
IQR	Interquartile range
LEDD	Levodopa equivalent daily dose
MDS-UPDRS	Movement Disorder Society–Unified Parkinson’s Disease Rating Scale
MMSE	Mini-Mental State Examination
MoCA	Montreal Cognitive Assessment
MS	Multiple sclerosis
NMS	Non-motor symptoms
NMS-Quest	Non-Motor Symptoms Questionnaire
NMSS	Non-Motor Symptoms Scale
OR	Odds ratio
PD	Parkinson’s disease
PwP/PwPs	Patient(s) with Parkinson’s disease
QUIP	Questionnaire for Impulsive-Compulsive Disorders in Parkinson’s Disease
R	Spearman’s rank correlation coefficient
ROS	Reactive oxygen species
SD	Standard deviation
UA	Uric acid
UA/Cr	Uric acid to creatinine ratio
